# Coping-Style Behavior Identified by a Survey of Parent-of-Origin Effects in the Rat

**DOI:** 10.1534/g3.118.200489

**Published:** 2018-08-22

**Authors:** Carme Mont, Polinka Hernandez-Pliego, Toni Cañete, Ignasi Oliveras, Cristóbal Río-Álamos, Gloria Blázquez, Regina López-Aumatell, Esther Martínez-Membrives, Adolf Tobeña, Jonathan Flint, Alberto Fernández-Teruel, Richard Mott

**Affiliations:** * Wellcome Trust Centre for Human Genetics, University of Oxford, OX3 7BN, UK; † Medical Psychology Unit, Department of Psychiatry & Forensic Medicine, Institute of Neurosciences, Universidad Autónoma de Barcelona, 08193-Bellaterra, Barcelona, Spain; ‡ Fundació Bosch i Gimpera, Parc Científic de Barcelona, 08028, Spain; § Universitat Pompeu Fabra, Department of Experimental and Health Sciences, 08003 Barcelona, Spain; ** Center for Neurobehavioral Genetics, University of California Los Angeles, Los Angeles, California; †† Genetics Institute, University College London, London WC1E 6BT, UK

**Keywords:** Multiparent Advanced Generation Inter-Cross (MAGIC), MultiParental Populations, MPP

## Abstract

In this study we investigate the effects of parent of origin on complex traits in the laboratory rat, with a focus on coping style behavior in stressful situations. We develop theory, based on earlier work, to partition heritability into a component due to a combination of parent of origin, maternal, paternal and shared environment, and another component that estimates classical additive genetic variance. We use this theory to investigate the effects on heritability of the parental origin of alleles in 798 outbred heterogeneous stock rats across 199 complex traits. Parent-of-origin-like heritability was on average 2.7fold larger than classical additive heritability. Among the phenotypes with the most enhanced parent-of-origin heritability were 10 coping style behaviors, with average 3.2 fold heritability enrichment. To confirm these findings on coping behavior, and to eliminate the possibility that the parent of origin effects are due to confounding with shared environment, we performed a reciprocal F1 cross between the behaviorally divergent RHA and RLA rat strains. We observed parent-of-origin effects on F1 rat anxiety/coping-related behavior in the Elevated Zero Maze test. Our study is the first to assess genetic parent-of-origin effects in rats, and confirm earlier findings in mice that such effects influence coping and impulsive behavior, and suggest these effects might be significant in other mammals, including humans.

Parental genotypes do not necessarily make equal contributions to the phenotypes of their offspring (Barlow 1995). In placental mammals, around 100 transcriptional units are imprinted (Morison *et al.* 2005; Lawson *et al.* 2013), *i.e.*, mono-allelically expressed in a parent-of-origin specific manner. The identities of these genes and the parental origin of the silenced allele depend on the species, tissue and developmental stage, with a core set of imprinted genes common to most species. In humans, disruption of specific imprinted genes cause developmental syndromes (Ishida and Moore 2013), and while the genome-wide impact of parent-of-origin effects (PoE) on human complex traits—as distinct from these syndromes—is less clear, a handful of examples are known; for example PoE at the imprinted *DLK1-MEG3* locus associates with type I diabetes (Wallace *et al.* 2010).

More is understood about PoE in mice; their phenotypic impact, as assessed in controlled reciprocal crosses and in populations of mice in which the parental origin of alleles can be determined, suggests PoE can be considerable. In (Wolf *et al.* 2008), quantitative trait loci (QTL) for growth were mapped in an advanced intercross of mice, in which offspring and their parents were genotyped: a significant fraction of the phenotypic variance attributable to QTL was due to PoE. In (Mott *et al.* 2014), heritability for 100 diverse phenotypes in a heterogeneous stock of mice, again with genotyped parents, was estimated based on genomewide genetic similarity, and partitioned into variance components representing PoE and non-PoE. The PoE component was on average about twice the non-PoE component. Similarly, phenotyping of the offspring of reciprocal crosses between inbred strains of mice have revealed PoE on growth (Mott *et al.* 2014), behavior (Hager and Johnstone 2003) and gene expression, in addition to maps of mono-allelic expression as a function of developmental stage and tissue (Babak *et al.* 2015).

PoE is widespread in other species, although reports are less comprehensive. For instance, cross-bred cattle harbor PoE QTL (Imumorin *et al.* 2011). However, only weak evidence for PoE was observed in poultry (Rowe *et al.* 2009). PoE (but not necessarily imprinting) is observed in other organisms such as *Drosophila melanogaster* (Wittkopp *et al.* 2006) and *Caenorhabditis elegans* (Sha and Fire 2005), despite the absence of DNA methylation in either species. Both PoE and imprinting affect embryogenesis in flowering plants (García-Aguilar and Gillmor 2015), and extensive epigenetic effects on flowering and other phenotypes occur in *Arabidopsis thaliana* (Cortijo *et al.* 2014).

However, there are potential difficulties of confounding when estimating PoE, and which might lead to other effects such as shared environment or dominance being mis-interpreted as PoE. Both of the family-based mouse studies of PoE (Wolf *et al.* 2008; Mott *et al.* 2014) included siblings that are also littermates. Siblings share more alleles by descent than non-siblings, and this excess is driven by alleles shared by common parents (Mott *et al.* 2014). Thus shared environment (*e.g.*, maternal effects and cage effects) potentially inflates the variance attributed to PoE (although cage effects were first removed in the calculation of heritability in (Mott *et al.* 2014)). Dominance effects can also be confounded with PoE (Rowe *et al.* 2009). Carefully designed and replicated reciprocal cross experiments can be used to eliminate these confounders, and have confirmed that in general PoE are real, although undoubtedly dominance and environment also play significant roles in the architecture of complex traits.

Furthermore, while the studies (Wolf *et al.* 2008; Mott *et al.* 2014) link genetic differences with PoE, they do not suggest a mechanism, except that the PoE QTL they identified did not typically overlap with known imprinted genes (most of which cluster into about 13 imprinted loci (Edwards and Ferguson-Smith 2007)). Thus the plausible assumption that only variation *in cis* at imprinted loci causes phenotypic PoE - notwithstanding syndromes in humans due to mutations near imprinted genes (Ishida and Moore 2013) – is not true. Indeed, transcriptome analysis of reciprocal crosses in (Mott *et al.* 2014) suggests that genetic variation *in trans* perturbs mono-allelic expression of imprinted genes to cause phenotypic PoE. Similarly, physiological PoE (Curley *et al.* 2004; Garfield *et al.* 2011; Mott *et al.* 2014) occur in reciprocal crosses between knockouts (KO) of non-imprinted genes and wild-type mice with isogenic backgrounds. Both phenotypic PoE and parental effects were observed, often sex-specific.

In rats, prenatal glucocorticoid overexposure generates multigenerational epigenetic effects on weight and on DNA methylation, transmitted through both paternal and maternal pathways (Drake *et al.* 2011; Cartier *et al.* 2018). Similarly, a high-fat diet generates metabolic PoE (Chambers *et al.* 2016). These environmental epigenetic effects suggest it would be worthwhile to assess the impact of genetic variation on PoE in rats, which also exhibit more complex behaviors than mice.

Different evolutionary theories underlie behavioral *vs.* developmental PoE (Keverne and Curley 2008), so it is important to understand the prevalence of PoE across different phenotype classes and species. In rats, it is known that environmentally-induced stress can alter behavior via epigenetic mechanisms (Bilang-Bleuel *et al.* 2005); we therefore asked if rat behavior is influenced by PoE in general, and specifically how rats and mice compare in their PoE responses to coping and impulsivity. “Coping style” is a cluster of behavioral and physiological characteristics that are stable over time and consistent across situations. It is a fundamental trait that defines how—and how adaptively—an organism deals (or copes) with threatening/stressful situations. The behavioral responses of rats to stressful situations, such as freezing, fleeing, fighting, risk assessment or self-grooming, are constituents of their particular coping style (Coppens *et al.* 2010).

Here, we employ two experimental rat resources. We start with data from a rat heterogeneous stock previously analyzed to map classical additive QTL across a broad range of phenotypes, including behavior (Baud *et al.* 2013). We ask whether heritability of complex traits shows a similar breakdown to that seen in mice. We derive a new parameterization of the variance components model used to partition heritability into PoE-like effects (including true PoE, maternal, paternal and shared environment) and non-PoE, which has some advantages of interpretation to that presented previously in (Mott *et al.* 2014).

We then focus on rat behavior, to determine whether PoE influence coping style behaviors in a reciprocal cross between RLA-I and RHA-I Roman rat strains. These strains were selected to differ strongly in their coping styles. RHA-I rats are proactive copers: they predominantly show active responses, such as fleeing, but very little freezing, when facing stressful/threatening situations. Their RLA-I counterparts are passive/reactive copers: they show much more freezing and self-grooming responses (Escorihuela *et al.* 1999; Díaz-Morán *et al.* 2012). These strains are therefore ideal for studying the genetic basis of two-way shuttle box avoidance acquisition, classically conditioned fear/freezing and unconditioned anxiety in the elevated plus-maze and zero-maze (Fernández-Teruel *et al.* 2002; Johannesson *et al.* 2009; Baud *et al.* 2013). To our knowledge, this study is the first to investigate PoE in laboratory rats and shows that, just as in mice, these effects are not negligible.

## Materials and Methods

### Theoretical Development

#### Parent-of-origin heritability in the HS rats:

The variance decomposition used in (Mott *et al.* 2014) separates the total phenotypic variance $\sigma^{2}$ into three parts, namely $\sigma_{+}^{2}$ associated with allele-sharing from parents of the same sex (and interpretable as due in part to parent-of-origin, maternal and paternal effects, and potentially shared environment when siblings are co-housed), $\sigma_{-}^{2}$ associated with allele-sharing from parents of the opposite sex (*i.e.*, the additive genetic variance not attributable to PoE), and $\sigma_{e}^{2}$, the environmental variance. Here we show this decomposition can be written in a mathematically equivalent way that makes its biological interpretation clearer. Earlier work on partitioning kinship matrices into maternal and paternal components is described in (Rowe *et al.* 2009, 2011).

First note that each genotype of a diploid individual can be split into maternally and paternally inherited components.  In a population sample of n individuals genotyped at l loci, these can be represented as a pair of n×l matrices M,P respectively, such that the i,k element is the (appropriately normalized) maternally or paternally inherited allelic dosage of individual i and locus k. The total genotype dosage matrix G=M+P. If the genotypes are all biallelic SNPs, and the SNP k is in Hardy-Weinberg equilibrium (HWE) with allele frequency $\pi_{k}$ then the un-normalized genotype dosages decompose into their maternal and paternal components as $g_{ik}=m_{ik}+p_{ik}$. The normalized genotype dosage matrix $G_{ik}$ decomposes into normalized maternal and paternal components thus:

$$G_{ik}=\frac{\left(g_{ik}-2\pi_{n}\right)}{\sqrt{2l\pi_{k}(1-\pi_{k})}}=\frac{\left(m_{ik}-\pi_{k}\right)}{\sqrt{2l\pi_{k}(1-\pi_{k})}}+\frac{\left(p_{ik}-\pi_{k}\right)}{\sqrt{2l\pi_{k}(1-\pi_{k})}}=M_{ik}+P_{ik}$$

If the SNPs are not in HWE then the sample means and standard deviations at each SNP are used to normalize the dosages. If the genetic markers are multi-allelic, or represent local haplotypes, as is the case in this study, then the definitions are slightly different: we use matrices defined in terms of the probability that an individual carries particular maternally inherited and paternally inherited haplotypes at a given locus. The formulae are given in (Mott *et al.* 2014) and are not repeated here (the decomposition for SNPs given here is new). The important point is that matrices M,P can be constructed, representing maternal and paternal genotype or haplotype contributions as required.

The additive genetic relationship matrix (GRM) K, of dimension n×n, that is routinely used to estimate heritability is usually defined as (Yang *et al.* 2011):

$$K=GG^{T}=\left(M+P\right){\left(M+P\right)}^{T}\;$$

(where the superscript T indicates matrix transposition). It can be partitioned thus:

$$K=(MM^{T}+PP^{T})+(PM^{T}+MP^{T})=K_{+}+K_{-}$$

Note that by construction the expected values of the diagonal elements of the standard symmetric GRM K are all unity, and the off-diagonal element $K_{ij}$ is the correlation between individuals i,j based on their normalized genotypes. The matrix element $K_{+ij}$ is the component of the genetic relationship between i,j that is attributable to allele-sharing from parents of the same sex. Thus if i,j are siblings this means inheritance from the same individual parent (*i.e.*, identity by descent), otherwise inheritance of the identical allele but from distinct individuals of the same sex (*i.e.*, identity by state and inherited from a parent of the same sex). The matrix $K_{-ij}$ is the component of allele-sharing in which the parents transmitting the shared allele are of opposite sex. Since the corresponding diagonal elements of $K_{+},K_{-}$ are all positive and sum to unity in expectation, individually they are less than unity.

The variance matrix of a Multivariate-Normally distributed phenotype z is modeled as a sum of these matrices, plus a diagonal matrix of uncorrelated environmental effects:

$$\;V(\sigma_{+}^{2},\sigma_{-}^{2},\sigma_{e}^{2})=K_{+}\sigma_{+}^{2}+K_{-}\sigma_{-}^{2}+I\sigma_{e}^{2}$$

Under the null hypothesis that there are no PoE then $\sigma_{+}^{2}=\sigma_{-}^{2}$ and the variance matrix collapses to the simpler and more familiar form

$$V(\sigma_{g}^{2},\sigma_{e}^{2})=K\sigma_{g}^{2}+I\sigma_{e}^{2}$$

where $\sigma_{g}^{2}$ is the usual additive genetic variance.

#### Reparameterisation:

It is desirable that each component of the variance decomposition should itself be a variance component, that is both V and each of the individual matrices in the decomposition should be positive semidefinite (PSD). By construction, both K and $\;K_{+}$, are positive-semidefinite (PSD) matrices since for any vector z,

$$z^{T}Kz=z^{T}GG^{T}z=\;{\left(G^{T}z\right)}^{T}\left(G^{T}z\right)\geq 0\;$$

and

$$\;\;\;\;\;\;\;\;\;\;\;\;z^{T}K_{+}z=z^{T}MM^{T}z+z^{T}PP^{T}z={\left(M^{T}z\right)}^{T}\left(M^{T}z\right)+{\left(P^{T}z\right)}^{T}(P^{T}z)\geq 0$$

However, $K_{-}$ is not of the same form and is not necessarily PSD. We provide here a reparameterisation that solves this difficulty and provides an alternative interpretation of the decomposition. First note that the log-likelihood of the data satisfies

$$\begin{array}{l} -2\log L(\sigma_{+}^{2},\sigma_{-}^{2},\sigma_{e}^{2})={\left(z-E(z)\right)}^{T}V^{-1}\left(z-E(z)\right)+\log|V|\\ +\;\text{const} \end{array}$$

which depends on $\sigma_{+}^{2},\sigma_{-}^{2},\sigma_{e}^{2}\;$solely through V, so any re-parameterisation that preserves V will also preserve the likelihood, and so have equivalent maximum-likelihood estimates. Define $\delta^{2}=\sigma_{+}^{2}-\sigma_{-}^{2}$ and reparameterise the variance as

$$V(\delta^{2},\sigma_{-}^{2},\sigma_{e}^{2})=K_{+}\delta^{2}+K\sigma_{-}^{2}+I\sigma_{e}^{2}$$

so that each component is PSD. Under the null hypothesis of no PoE, $\delta^{2}=0$ and the variance becomes that of the standard additive genetic model. The valid parameter space for this variance component is $\delta^{2}\geq 0$, *i.e.*, $\sigma_{+}^{2}\geq \sigma_{-}^{2}$. In the region $\delta^{2}>0$ the likelihoods are equivalent under both parameterisations so the maximum likelihood estimates (MLEs) satisfy $\hat{\delta^{2}}=\hat{\sigma_{+}^{2}}-\hat{\sigma_{-}^{2}}$. However, if $\hat{\delta^{2}}<0$ then $\hat{\delta^{2}}$ will be set to zero (*i.e.*, moved to the boundary) and the two parameterisations will report different MLEs. These observations also apply to the heritabilities $h_{+}^{2}$, $h_{-}^{2},\;h_{\delta^{2}}^{2}$ associated with $\sigma_{+}^{2},\;\sigma_{-}^{2},\;\delta^{2}$ respectively, each computed by dividing by the total phenotypic variance.

This reparameterization also shows, irrespective of the truth of the null hypothesis, that $\sigma_{g}^{2}=\sigma_{-}^{2}$. In other words, $\sigma_{-}^{2}$ is an estimator of the additive heritability $\sigma_{g}^{2}\;$that would be estimated in population samples without any first-degree relatives (*e.g.*, in genetic association studies). Thus, even in a population sample containing siblings, provided the parents are unrelated, the variance matrix $K_{-}=MP^{T}+PM^{T}\;$is an unbiased estimator of the matrix 0.5K that would be obtained from a sample of unrelated individuals, in the sense that

E(M)=E(P)=0.5E(G)

so that, because M,P are independent,

$$E\left(K_{-}\right)=E\left(M^{T}P+P^{T}M\right)=0.5E(K)$$

Thus $\sigma_{-}^{2}$ estimated from related individuals equals the additive heritability that would have been observed in a sample of unrelated individuals. In contrast, $\sigma_{+}^{2}$ is the additive heritability, possibly inflated by family relatedness should the null hypothesis be false. The excess heritability $\delta^{2}$ can be caused by PoE, but may also be confounded by shared environment, for example maternal effects.

One can then make inferences about genetic architecture via hypothesis tests on the m.l.e.s of $\sigma_{+}^{2}$,$\;\sigma_{-}^{2},\sigma_{e}^{2}$, using the information matrix to generate variance and covariance estimates.

It is worth noting that the likelihood is well defined whenever the overall variance matrix V is positive definite (PD), which is the case even for small but negative values of $\delta^{2}$. Since $I\sigma_{e}^{2}$ is always positive definite, a sufficient condition that V is positive definite is that $K_{+}\sigma_{+}^{2}+K_{-}\sigma_{-}^{2}$ also be positive definite. Since both $K=K_{+}+K_{-}$ and $K_{+}$ are positive definite, and

$$K_{+}\sigma_{+}^{2}+K_{-}\sigma_{-}^{2}=K_{+}\sigma_{+}^{2}+(K-K_{+})\sigma_{-}^{2}=K_{+}\left(\sigma_{+}^{2}-\sigma_{-}^{2}\right)+K\sigma_{-}^{2},$$

it follows that V is positive definite whenever $\sigma_{+}^{2}-\sigma_{-}^{2}\geq 0$, which is precisely the region of interest in a PoE analysis. In fact, provided $\sigma_{e}^{2}>0$, the region for which V is positive definite will also include some $\sigma_{+}^{2}<\sigma_{-}^{2}$, the boundary depending on $K_{+},K_{-}$ and on the phenotype vector in question. However, there do not appear to be biologically realistic scenarios for which $\sigma_{+}^{2}<\sigma_{-}^{2}.$ Under the null hypothesis that there is no PoE then $\sigma_{+}^{2}=\sigma_{-}^{2}.$ This can be thought of as being an edge of the parameter space.

Finally, partitioning the variance into $\sigma_{+}^{2},\sigma_{-}^{2},\sigma_{e}^{2}$ differs from spatial partitioning, such as when each chromosome has a unique GRM and heritability (Yang *et al.* 2011), or when the genome is divided according to feature annotation (Speed *et al.* 2012). It more closely resembles the approach in (Zaitlen *et al.* 2013), in which a GRM approximating the IBD degree of genetic relatedness expected due to the pedigree is effectively subtracted from K to generate approximate analogs of the matrices $K,K_{+}$, used here. The details of the matrix construction in (Zaitlen *et al.* 2013) are different, however.

### Data

#### HS Rats:

The HS rat data used in this study are a subset of that reported in (López-Aumatell *et al.* 2011; Baud *et al.* 2013; Baud *et al.* 2014). Genotypic data were available for 798 individuals, for which both parents were genotyped (198 parents) at 265,551 SNPs using the RATDIV SNP array. Phenotypes for 199 traits were available for subsets ranging from to 205 to 617 individuals (Supplemental Table S1). Estimates of heritability were obtained according to (Mott *et al.* 2014) (Methods). We used the R package happy.hbrem (Mott *et al.* 2000) to compute phased haplotype probabilities, from which kinship matrices were computed and analyzed. Variance components $\sigma_{+}^{2},\sigma_{-}^{2},\sigma_{e}^{2}$ were computed using GCTA (Yang *et al.* 2011) applied to these Genetic Relationship Matrices (GRM) $\;K_{+},K_{-}$. Analyses were performed in R (R Core Team 2018).

#### RHA/RLA Roman Rats F1 reciprocal cross:

The Wistar-derived outbred sublines of Roman High-(RHA/Verh) and Low-(RLA/Verh) Avoidance rats have been genetically selected since 1972 based on their good (RHA/Verh) *vs.* extremely poor (RLA/Verh) acquisition of two-way, active avoidance (Driscoll *et al.* 1998; Steimer and Driscoll 2003). The inbred strains RHA-I and RLA-I, derived from those two lines, have been maintained at our laboratory in the Autonomous University of Barcelona since 1996 (Driscoll *et al.* 1998; Escorihuela *et al.* 1999).

Reciprocal crosses between the 60^th^ generation of RHA-I and the RLA-I inbred rat strains (hereafter RHA and RLA, respectively) were set up with the following configuration: female RHA/male RLA (fRHA/mRLA) and female RLA/male RHA (fRLA/mRHA). The breeding pairs were housed together and separated once pregnancy was confirmed. Animals were tested from the F1 crosses, comprising 34 fRHA/mRLA pups (19 females and 15 males) and 37 fRLA/mRHA pups (22 females and 15 males). Pups were weaned and caged in pairs of siblings in macrolon cages (50 cm × 25 cm × 14 cm). They were maintained with food and tap water available *ad libitum*, under controlled temperature (22° ± 2°; 50–70% humidity) and a 12-h light/12-h dark cycle (lights on at 08:00 h). Behavioral testing commenced at 8-10 weeks of age, with one-week separation between the two tests. Experiments were performed at the Medical Psychology Unit, Department of Psychiatry & Forensic Medicine, Autonomous University of Barcelona, Spain, between 09:00 and 19:00h in accordance with the Spanish legislation on “Protection of Animals Used for Experimental and Other Scientific Purposes” and the European Communities Council Directive (86/609/EEC) on this subject.

#### Elevated Zero Maze:

The maze comprised an annular platform (105 cm diameter; 10 cm in width) made of black plywood and elevated to 65 cm above the ground level. It had two open sections (quadrants) and two enclosed ones (walls 40 cm high). The subject was placed in an enclosed section facing the wall. The apparatus was situated in a black testing room, dimly illuminated with red fluorescent light, and the behavior was videotaped and measured outside the testing room by an expert observer who was blind to the cross condition. Latency to enter into an open section, time spent in the open sections, number of entries in the open sections, number of head dips (through the edge of the open sections), number of line crossings and number of stretched attend postures (SAP, from a closed to an open section of the maze) were measured for 5 min. All these measures (except “line crossings”, which are an index of overall locomotor activity), have been pharmacologically and behaviorally validated as anxiety-related parameters (Shepherd *et al.* 1994; Lopez-Aumatell *et al.* 2008, López-Aumatell *et al.* 2009).

#### Two-way active shuttle-box avoidance acquisition:

Active avoidance acquisition sessions were performed in three identical shuttle boxes (Letica Instruments), each one placed in independent sound-attenuating boxes consisting of two equal-sized compartments (25 × 25 × 28 cm) connected by an opening (8 × 10 cm) and illuminated by a dim fluorescent light (<50 lux). Rats were allowed a 4 min period of familiarization to the box. Immediately after that period, a 40-trial session/rat was administered, each trial consisting of a 10 s CS (conditioned stimulus; 2400 Hz, 63-dB tone plus a 7 W small light) followed after termination by a 20 s US (unconditioned stimulus; scrambled 0.7-mA foot shock) delivered through the grid floor (López-Aumatell *et al.* 2009). Crossings to the other compartment during the CS (avoidances) or US (escapes) switched off the stimuli and were followed by a 60 s inter-trial interval. Avoidances, escapes, latency to escape/avoid, inter-trial crossings (crossing to the other compartment during the inter-trial period) and context-freezing (complete and rigid immobility –except for breathing movements- during the first 5 inter-trial intervals of the avoidance training session) measurements were recorded. Freezing was measured by an expert observer who was blind to the cross direction.

These parameters (in particular, avoidances, latency to escape/avoid, inter-trial crossings and context-conditioned freezing) have been pharmacologically and behaviorally validated as anxiety/fear- and coping style-related measures (Fernández-Teruel *et al.* 1991; Driscoll *et al.* 1998; Steimer and Driscoll 2003; López-Aumatell *et al.* 2011; Vicens-Costa *et al.* 2011; Díaz-Morán *et al.* 2012). The data were detected and loaded into a computer automatically, except the context-freezing which was measured by a researcher during the first five inter-trial intervals.

#### Statistical Analysis of behavior:

F1 rat phenotypes were analyzed using R. First, a principal components analysis identified a family with extreme values and three subjects of the family were eliminated from the sample for all the measures. The remaining behavioral data were analyzed by the R function lmer() in the R package lme4 (Bates *et al.* 2015). Analyses fitted family (defined as the concatenation of maternal and paternal id) as a random effect and parental origin (encoded by the identity of the maternal strain, mstrain), plus sex as fixed effects (Supplemental Tables S2, S3). For example the trait freezing5 would be modeled in R as

lmer(freezing5∼sex + mstrain + (1|Family))

Significance of fixed effects was determined by analysis of deviance with the R command anova(), using the chi-squared distribution on 1 degree of to compute P-values of the PoE effect (*i.e.*, whether the fixed effect parameter estimate associated with mstrain was significantly different from zero).

### Data Availability

HS phenotypes and genotypes are downloadable from Baud *et al.* 2014. Phenotypes for reciprocal cross are provided in Supplemental Tables S2, S3. Supplemental material available at Figshare: https://doi.org/10.25387/g3.6587681.

## Results

### Heritability and parent of origin effects in HS rats

The chromosomes of the HS rats are highly recombined mosaics of the eight progenitor haplotypes of the HS. Following (Mott *et al.* 2014), we define a PoE at a locus as a difference in the phenotypic effect due to an HS progenitor haplotype, depending on whether it originated in the previous generation from the father or mother. To do this, we computed the phased probabilities that an animal inherits the ancestral founder haplotype from each parent at each locus, and then used these probabilities to partition the kinship by PoE.

**Figure 1:**
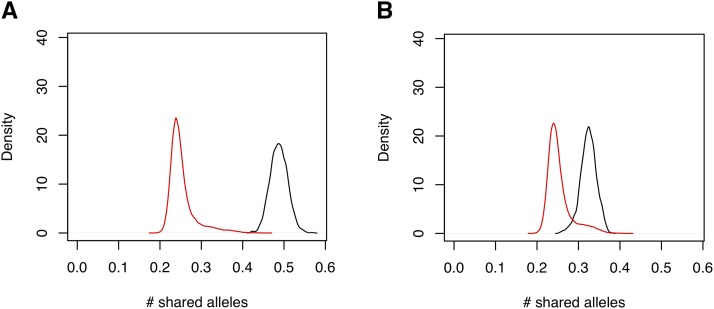
Frequency distributions of the off-diagonal elements of the Genetic Relationship Matrices $K_{+}$ (A) and $K_{-}$ (B) in HS rats. In each plot, the distributions of siblings (black) and non-siblings (red) are shown separately; x-axis shows the number of shared alleles, y-axis is the smoothed density.

Parental genotypes were available for 798 HS rats, allowing genotypes to be phased with respect to parental origin. A list of the phenotypes and number of rats phenotyped for each trait is described in Supplemental Table S1. Using 265,551 single nucleotide polymorphisms (SNPs) to estimate founder haplotypes at each locus and individual, we derived a genetic relationship matrix K which was partitioned into components representing allele sharing from parents of the same sex $K_{+}$, and from parents of the opposite sex,$\;K_{-}$. Figure 1 plots the distributions of the entries of these matrices, and shows that, consistent with previous work in HS mice, in $K_{+}$ (Figure 1A) the siblings have distinctly more allele-sharing than non-siblings. In $\;K_{-}$ (Figure 1B) the distributions are closer together, but interestingly - and in contrast to HS mice - they are still separable. Figure 1B shows that rats selected for mating were more closely related than would be expected if they were chosen at random. If parents are unrelated then the expected distribution of $K_{-}$ for siblings and non-siblings should be equal, as was observed for HS mice (Mott *et al.* 2014) (Table 1).

**Table 1:** P-values for the effects of sex and parent-of-origin (PoE) for coping-style behavior as measured in 68 rats from a reciprocal cross of RHA and RLA strains, in the elevated zero-maze (zero) and shuttle box (shuttle). Phenotypes are: zlatency (time/s to first entry into the open sections of the zero maze), timeopen (time/s spent in open sections of the maze), entries (number of entries into maze open sections), Head_Dips (number of head dips through the edge of the open sections of the maze), SAP (number of stretch attend postures from a closed to an open section of the maze), LineX (number of line crossings in the elevated zero maze), Freezing5 (context freezing, *i.e.*, time spent freezing during the first 5 inter-trial intervals of the two-way avoidance –shuttle box- training session), avoid (number of avoidances during the 40-trial avoidance session), latency (time/s to escape, averaged for the 40 training trials of the avoidance session), ittx (inter trial crossings during the forty 60-min inter-trial intervals of the avoidance session). P-values are calculated from a mixed-model analysis of deviance, *i.e.*, from chi-squared statistics on one degree of freedom.

test	phenotype	sex	PoE
zero	zlatency	0.12930	0.14979
zero	timeopen	0.15453	0.00306
zero	entries	0.27875	0.00367
zero	Head_Dips	0.75750	0.17803
zero	SAP	0.00075	0.40865
zero	LineX	0.63659	0.01728
shuttle	Freezing5	0.10342	0.11651
shuttle	avoid	0.86100	0.78989
shuttle	latency	0.28069	0.50258
shuttle	ittx	0.12568	0.56464

**Figure 2:**
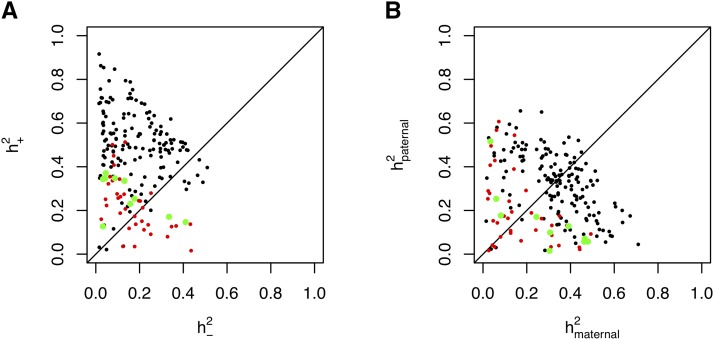
(A) Heritability of 199 traits partitioned into components $h_{+}^{2}$ (y-axis) and $h_{-}^{2}$ (x-axis) associated respectively with PoE and non-PoE. Each dot represents one trait. Coping-Style traits are in green, other behavioral traits are in red. (B) Heritability partitioned into maternal and paternal components, color-coded as in (A).

Next, for each of the 199 phenotypes we used a mixed model (Yang *et al.* 2011) to estimate the fraction of phenotypic variation attributable to each component of inheritance $h_{+}^{2}$ and $h_{-}^{2}$. If the parent of origin makes no contribution then we expect $h_{+}^{2}=h_{-}^{2}$. In 86% of the traits examined (172 out of 199), $h_{+}^{2}>h_{-}^{2}$ (Figure 2A). The medians are 0.474 and 0.155 respectively with a median ratio $h_{+}^{2}/h_{-}^{2}=2.66.$ The average standard errors are 0.133 for $h_{+}^{2}$ and 0.172 for $h_{-}^{2}$. The heritability attributable to $h_{+}^{2}$ and $h_{-}^{2}$ and the total heritability for each of the phenotypes, is detailed in Supplementary Table S1. The numbers of rats for many phenotypes was too small to attempt to map PoE QTL.

In the Methods we show this model for PoE and non-PoE heritability can be re-parameterised in terms of $\delta^{2}=\;h_{+}^{2}-h_{-}^{2}$ and $h_{-}^{2}$. The reparameterisation makes biological interpretation more straightforward: $\delta^{2}$ is the additional genetic variance explained by PoE and $h_{-}^{2}$ is what would be observed in a sample of unrelated individuals whose parents were also unrelated; in other words $h_{-}^{2}$ is an estimate of the classical additive heritability.

Maternal effects can be confounded with PoE (Hager *et al.* 2008). Therefore we examined whether the phenotypes showed a higher maternal heritability. For all the 199 traits, the median maternal heritability is 0.323 with a standard error of 0.165, and 0.295 for the paternal heritability with a standard error of 0.164. The median ratio of maternal heritability/paternal heritability = 1.08 (Figure 2B, Supplemental Table S1). We thus find little evidence that maternal effects play a larger role than paternal effects. A recent study of paternally transmitted epigenetic effects on rats exposed to glucocorticoids supports this general observation (Cartier *et al.* 2018).

### Parent of origin effects contribute to coping-style behavioral phenotypes

We analyzed 45 behavioral measures, including phenotypes from the elevated zero maze, the novel-cage activity test, context-conditioned freezing and the two-way active avoidance test. We found that these measures had lower than average $h_{+-}^{2}$ values (Figure 2A). In only 57% of the behavioral traits examined (26 out of 45 measures), $h_{+}^{2}>h_{-}^{2}$. A binomial test of the null hypothesis that the percentage should be 50% was not significant (*P* = 0.116). The average standard errors of the heritability estimates were 0.122 and 0.171 respectively. The medians were $h_{+}^{2}=0.242$ and $h_{-}^{2}=0.131$ with a median ratio of $h_{+}^{2}/h_{-}^{2}=1.68$. This contrasts to the ten coping-style behaviors (listed in Supplementary Table S1), In eight out of the ten measures $h_{+}^{2}>h_{-}^{2}$ (binomial *P* = 0.011, Figure 2A; we note that the binomial test may be anti-conservative due to phenotypic correlations). The medians are $h_{+}^{2}=0.294$ and $h_{-}^{2}=0.109$ with a median ratio of $h_{+}^{2}/h_{-}^{2}=3.25$ and standard errors of 0.137 and 0.193 respectively. Thus in general behavioral traits show less evidence of PoE, except for coping-style behaviors which exhibit stronger PoE than do most other traits.

### Reciprocal crosses between RHA-RLA strains show differences in coping style depending on parental origin

We confirmed our observations on coping style behaviors in the HS by testing for PoE in a reciprocal cross. As noted above, RHA and RLA are inbred rat strains derived from the Wistar stock of rats in an experiment that selected for differences in coping behavior (*i.e.*, acquisition of the two-way active avoidance response). RLA rats show increased stress-induced endocrine responses, enhanced anxiety/fearfulness and passive/reactive coping style in a variety of unconditioned behavioral variables and tests, compared to RHA (Driscoll *et al.* 1998; Steimer and Driscoll 2003; Díaz-Morán *et al.* 2012). We therefore chose these strains for behavioral testing in preference to picking two founders of the HS, in order to increase the power to detect parent of origin effects on coping behavior.

We made reciprocal F1 crosses (female RHA x male RLA (fRHA/mRLA) or female RLA x male RHA (fRLA/mRHA) and produced 137 offspring in total. The mRHA/fRLA was more fertile than the other cross (103 pups *vs.* 34) so we phenotyped offspring at random to balance the numbers of families, offspring sex and totals for the two cross directions. We measured behavioral responses in the elevated zero maze and the two-way active avoidance in the shuttle box. In total phenotypes from 68 offspring from 19 families were analyzed.

All rats within a family have the same parental origin of alleles. Therefore, to eliminate possible confounding between families and PoE, we fitted a mixed model in which membership across the 19 families was treated as a random effect, while cross direction (parent of origin) and sex were fixed effects. Controlling for family membership in this way enables the testing for parent of origin effects and reduces the possibility of a false positive result due to random differences between familial environments. To test for PoE in the mixed model we subtracted the log-likelihood for a null model from a PoE model, resulting in a chi-squared statistic with one degree of freedom.

**Figure 3:**
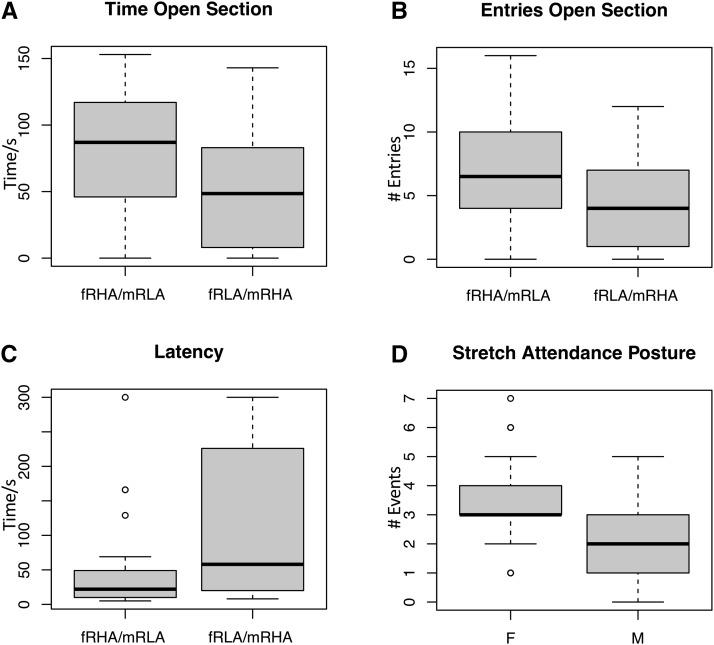
Box-and-whisker plots showing parent-of-origin effects on three behaviors (A-C) and gender effects on Stretch Attendance Posture (D), in 68 F1 rats from a reciprocal cross between strains RHA and RLA. x-axis indicates direction of reciprocal cross (fRHA/mRLA *vs.* fRLA/mRHA) in A-C and sex (M:Male, F:Female) in D. y-axis is behavioral phenotype score (A: Time in Open Section/s, B: number of Entries into Open Section, C: Latency to enter into an open section/s, in Elevated Zero Maze, D: Number of stretch attendance postures (SAP) in Elevated Zero Maze). Thick black horizontal line indicates median, gray box indicates range of central 50% of data points, and outer whiskers the 90% range.

In the elevated zero maze, fRHA/mRLA rats enter the open section earlier, make a higher number of entries, explore more quadrants of the maze, and remain in the open section for longer, showing an active coping style similar as the RHA rat strain (Figure 3A-C). fRLA/mRHA rats show the opposite behavioral response, characteristic of a reactive coping style and similar to the RLA strain. For these measures, the behavioral profile of the fRHA/mRLA and fRLA/mRHA rats is more similar to their maternal strain than to their paternal strain.

In the elevated zero maze there were no significant differences between fRHA/mRLA or fRLA/mRHA rats in numbers of head dips or defecation, irrespective of gender. Gender was significant in stretch attend postures (SAP); females performed significantly more SAP (*P* = 0.00075) (Figure 3D), but there was no SAP effect for PoE (*P* = 0.409) and no significant interaction between these factors. There were significant PoE for time spent in the open sections of the maze (*P* = 0.00306), the number of entries (*P* = 0.00367) and the number of line crossings (*P* = 0.01728).

In a two-way active avoidance in the shuttle box no significant differences between the fRHA/mRLA and fRLA/mRHA rats were observed for escape/avoidance latency, inter trial crossings, number of avoidances and context-freezing. There was also no gender effect or interaction between gender and parental origin.

## Discussion

In this study we have surveyed the heritability in HS rats of 199 complex traits, 86% of which show an apparent PoE. Our results are consistent with those observed previously in HS mice, in which we also identified pervasive PoE in the heritability of complex traits (Mott *et al.* 2014). In HS mice, the enrichment for PoE, as measured by the median ratio of $h_{+}^{2}/h_{-}^{2}$ was smaller than reported here in HS rats ($h_{+}^{2}/h_{-}^{2}=2.04$ *vs$\;\;h_{+}^{2}/h_{-}^{2}=2.66$* respectively). However, the smaller HS rat sample means that the estimates have larger standard errors (average 0.133 for $h_{+}^{2}$ and 0.172 for $h_{-}^{2}$ in rats compared to 0.058 and 0.078 in mice). We did not attempt to map PoE QTL in this study, because of low power due to the relatively small sample size. These results are based on genome-wide genetic similarities, and so are unlikely to be driven entirely by variation at imprinted genes. While confounding with shared environment might explain some of the excess heritability, our results suggest that PoE also originate from loci that are not imprinted. In addition to shared environment, dominance can also generate apparent PoE (Rowe *et al.* 2009). It is therefore important to verify the PoE we detected by heritability analysis of HS using an orthogonal experimental design—such as reciprocal cross—as we have done here.

There are two differences between the mouse and rat HS experiments requiring comment. First, the HS mice were housed in cages with 4-5 same-sex littermates. The HS rats were housed in cages with just two same-sex littermates. This meant that in the mice, it was possible to remove cage effects before estimating heritability, but in the rats it was not possible to do this as they were too closely confounded. Consequently the estimates of PoE heritability in the rats are likely to be inflated by shared environment. Second, the rats were more closely related than the mice in the sense that many of the parents were cousins. This alters the distribution of allele sharing attributed to parents of opposite sex ($K_{-}$, Figure 1B) and makes the distributions of $K_{+}$, $K_{-}$, more alike in the rats than the mice, thereby potentially reducing our power to detect differences between PoE and non-PoE heritability.

The phenotypes obtained from the HS rats included a large battery of behavioral measures. These included avoidance acquisition in the shuttle box and context-conditioned freezing (Vicens-Costa *et al.* 2011) both of which assess coping style, and which is also predicted by and correlated with latency to the first entry into an open section and entries to the open section of the elevated zero maze (Fernández-Teruel *et al.* 1991; Escorihuela *et al.* 1999; Lopez-Aumatell *et al.* 2008, López-Aumatell *et al.* 2009; Vicens-Costa *et al.* 2011; Díaz-Morán *et al.* 2012; Coppens *et al.* 2013).

We observed PoE in 8/10 coping style behaviors in HS rats, which we confirmed in a reciprocal cross of the Roman low- and high-avoidance rats. These strains exhibit opposite responses to stress (Driscoll *et al.* 1998): RLA and RHA rats have respectively reactive and pro-active coping styles (Steimer and Driscoll 2003; Díaz-Morán *et al.* 2012; Coppens *et al.* 2013), while HS rats have a profile similar to the RLA rats in their coping style and responses to stress (López-Aumatell *et al.* 2009; Díaz-Morán *et al.* 2012). We found F1 rats with RHA fathers and RLA mothers behaved in the zero maze with an RLA-like reactive coping style (as assessed by previous tests of RLA and RHA behavior), while those with RLA fathers and RHA mothers more closely resembled RHA rats. These differences remained significant even after removing litter effects, so having a phenotypic response linked to the parental strain cannot be attributed solely to shared environment or maternal effects.

Epigenetic modifications in the serotonin and glutamate receptors within in the prefrontal cortex differ between RLA and RHA rats, supporting the general principle that PoE might affect behavior via epigenetic effects in these strains (Fomsgaard *et al.* 2018). Besides differing in coping style, RHA and RLA rats exhibit different impulsivity, behavioral inhibition and behavioral flexibility. Thus RHA *vs.* RLA differences align with those in reciprocal crosses of mice in which the imprinted genes *Gbr10* or *Nesp55* are knocked out (Dent *et al.* 2016, 2018).

As is thought to occur in humans with respect to similar traits (Coppens *et al.* 2010; Jupp *et al.* 2013), RHA rats present functional deficits in the prefrontal cortex and hippocampus and amygdala, three very closely linked structures that are involved in coping, impulsivity, behavioral flexibility and behavioral inhibition. They also present differences in the serotoninergic system (and other neurotransmitters) with respect to RLA rats. Our results therefore suggest that it would be worthwhile to investigate the impact of PoE on human behavior.
